# Post lung-transplant predictive value of thermodilution vs estimated Fick cardiac output measurement

**DOI:** 10.1016/j.jhlto.2025.100228

**Published:** 2025-02-14

**Authors:** Jose Rivera-Robles, Komal Alam, Ahmed Abdelmonem, Audrene Edwards, Ahmad Abdelreheim, Susan K. Mathai, Michael Duncan, Chetan Naik

**Affiliations:** a Baylor University Medical Center, Dallas, Texas; b Texas A&M University, College of Medicine, Bryan, Texas

**Keywords:** pulmonary hypertension, cardiac output, Fick, lung transplant, thermodilution

## Abstract

**Background:**

Comparison of thermodilution (TD) and indirect Fick (iFick) methods of cardiac output (CO) measurement has not been well described in patients with World Health Organization (WHO) group 3 pulmonary hypertension (PH).

**Methods:**

We conducted a single-center retrospective chart review of 96 patients with WHO group 3 PH who underwent lung transplantation. For comparison, 32 WHO group 1 pulmonary arterial hypertension patients who were followed in our PH clinic during the same period were also included in the study.

**Results:**

TThere was a significant difference between iFick CO and TD CO (5.93+/ -1.5 versus 5.46+/ -1.8 liter/minute, *p*=0.0061) in WHO group 3 PH. Pulmonary vascular resistance (PVR) calculated using iFick and TD–CO values also differed significantly. TD–PVR was more strongly associated with measures of poor outcomes after lung transplant.

**Conclusions:**

iFick-CO and TD-CO can be significantly different in WHO group 3 PH. In cases of discrepancy between iFick and TD-COs, TD-CO correlates better with clinical outcomes after lung transplantation.

Pulmonary hypertension (PH) is a disorder characterized by elevated pulmonary arterial pressures; if left untreated, it is associated with high morbidity and mortality. PH is defined as an elevation of mean pulmonary arterial pressure (mPAP) greater than 20 mm Hg measured during right heart catheterization (RHC).^1^ Cardiac index (CI), right atrial pressure, and mixed venous oxygen saturation values obtained during RHC are also helpful in prognosticating and risk stratification of PH.^2^, ^3^ PH is classified into 5 categories based on the similarities in etiology, clinical presentation, pathological findings, hemodynamic characteristics, and treatment strategies.^4^ World Health Organization (WHO) group 3 PH is PH caused by lung diseases or hypoxia/hypoventilation. The pathogenesis of WHO group 3 PH is complex and is contributed to by the destruction of pulmonary parenchyma and also pulmonary arteriopathy similar to that seen in pulmonary arterial hypertension (PAH).^5^ Although PH develops in a minority of patients with chronic lung diseases, it is associated with severe symptoms and worse survival.^6^, ^7^, ^8^, ^9^, ^10^ Pulmonary vascular resistance (PVR) of >5 Wood units in patients with chronic lung diseases is associated with significantly worse survival.^11^, ^12^ Thus, PVR is an important calculated value that has diagnostic and prognostic implications.

The accuracy of PVR depends on the measurement of cardiac output (CO). Several invasive and noninvasive techniques have been used to measure CO in humans.^13^, ^14^ The “gold standard” is the direct Fick method in which the CO is calculated as the quotient of oxygen uptake per minute (VO_2_) and the difference of the arterial and mixed venous oxygen content. The direct Fick method is only rarely used in clinical practice because the bedside measurement of oxygen uptake is technically demanding. The indirect Fick (iFick) method has gained popularity as it offers the convenience of using calculated oxygen consumption by the Lafarge Miettinen equation.^16^ Since the introduction of the Swan-Ganz catheter, the thermodilution (TD) method has been used to measure CO during RHC.^15^ TD consists of injecting room temperature or cold 5% dextrose or saline and calculating the temperature fall and rise after the administration of the fluid. The mean decrease in temperature will be inversely proportional to the CO. Due to the ease and cost-effectiveness of iFick method and TD methods, they are commonly used techniques to measure CO with RHC reports frequently describing both values.

Several studies have reported discrepancies in CO measurements between these 3 methods. Most of these studies have focused on patients with WHO group 1 PH, and there is a dearth of studies comparing iFick and TD-CO measurement methods in patients with WHO group 3 PH. With the establishment of consensus opinion on criteria to trial vasodilators in severe WHO group 3 PH^1^ and the approval of inhaled treprostinil in treating PH in the setting of ILD^17^, the accuracy of measuring CO and PVR in this population is paramount. The presence of PH is also associated with worse post lung transplant outcomes, namely, perioperative right ventricular failure requiring a mechanical support device, a higher incidence of primary graft dysfunction (PGD), and increased mortality in the first year.^18^, ^19^, ^20^ Hence, patients with WHO group 3 PH who underwent lung transplants at our institution allowed us to compare the iFick and TD methods in these patients and correlate them with outcomes after lung transplantation.

## Methods

### Data source

We performed a retrospective cross-sectional study with up to 1 year of follow-up of subjects from the date of lung transplantation. The study was approved by the institutional review board (IRB) at our institution (IRB Protocol 018-816). Consecutive patients with evidence of PH (mPAP > 20 mm Hg) who underwent lung transplantation between January 1, 2016, and December 31, 2021, were included in the study. A cohort of WHO group 1 PAH who were followed in the PH clinic during this duration were randomly selected for comparison. Patients were excluded if their RHC had missing data points (mPAP, iFick, or TD COs) and pulmonary arterial wedge pressure. The following data were collected post lung transplant for analysis: need for inhaled nitric oxide (iNO), PGD grade, number of days on mechanical ventilator, need for tracheostomy, total intensive care unit (ICU) stay, total length of hospital stay, and 1-, 3-, and 12-month mortality.

### Data analysis

#### Bivariate descriptive analysis

Sample characteristics are provided for the variables available to both WHO groups 1 and 3 using descriptive statistics. Categorical variables were described by frequencies and percentages. Means and standard deviations (or medians and ranges where appropriate) were used to describe continuous variables.

The bivariate analysis investigated differences between CO and CI, as measured by iFick and TD methods, among WHO group 3 PAH patients. The bivariate analysis used a paired *t*-test or Wilcoxon Signed-Rank Sum test serially to determine if there was a statistical significance in which group has more pronounced differences between the CO and CI. Statistical significance was assessed at a significance level of <0.05. All statistical analyses were performed using R (v. 4.2; R Foundation for Statistical Computing, Vienna, Austria).

#### Correlation analysis

The Wilcoxon Signed-Rank test was used to determine whether there are statistically significant differences between TD-PVR and iFick-PVR in the WHO group 3 patients.

#### Regression analysis

We studied the association/correlation of outcomes after lung transplant with the iFick-PVR and TD-PVR for the WHO group 3 PAH patients by using a regression analysis/point biserial correlation. The outcomes after lung transplant that were chosen for this analysis are (1) perioperative need for mechanical circulatory devices; (2) need for iNO; (3) need for cardiopulmonary bypass (CPB); (4) post-transplant PGD grade; (5) mechanical ventilator days; (6) ICU days; (7) total length of stay, and (8) 1-, 3-, and 12-month mortality.

#### Bland-Altman plot

To compare iFick-CO and TD-CO, Bland-Altman analysis was used to determine if there were any significant differences in discrepancy (bias or variability) between the 2 measurements. A Bland-Altman plot was used to graphically display the mean difference (bias), the 95% confidence interval within 2 standard deviations of the mean difference, and the Lin’s Concordance Correlation coefficient along with the 95% confidence interval was calculated to assess the relationship of the agreement between the 2 methods.

## Results

Our retrospective, cross-sectional study included 128 unique patients who fulfilled the inclusion criteria: 96 patients with WHO Group 3 PH and 32 patients with WHO Group 1 PH. As expected, patients with WHO group 3 PH were older, predominantly male, and had lower mPAP when compared to patients with WHO group 1 PH (Table 1). The median time from RHC to lung transplantation was 144 days (Interquartile range (IQR) 64-932).

**Table 1:** Patient Demographics

	WHO group 3 PH (*n* = 96)	WHO group 1 PH (*n* = 32)	*p* value
Age (years) median (IQR)	62 (8.0)	55 (16.5)	0.0003
Female %	32.3	68.8	0.0003
mPAP mm Hg (median (IQR)	26.5 (10.5)	43.5 (21.5)	<0.0001
PAWP mm Hg (median (IQR)	11 (8.5)	9 (7.5)	0.0303
CO liter/min (Fick) mean (SD)	5.92 (1.5)	4.77 (1.86)	0.0006
CI liter/min/min^2^ (Fick) mean (SD)	3.07 (0.78)	2.65 (1.24)	0.0269
CO (TD) liter/min mean (SD)	5.45 (1.27)	5.34 (1.64)	0.6929
CI liter/min/min^2^ (TD) mean (SD)	2.80 (0.65)	2.83 (0.90)	0.8479

CI, cardiac index; CO, cardiac output; iFick, indirect Fick; IQR, interquartile range; mPAP, mean pulmonary arterial pressures; PAWP, pulmonary arterial wedge pressure; PH, pulmonary hypertension TD, thermodilution; WHO, World Health Organization.

Parentheses indicate median or mean values.

**Figure 1:**
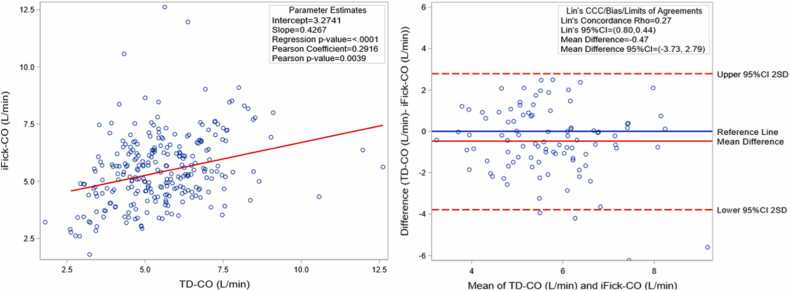
Regression and Bland-Altman analysis comparing iFick-CO and TD-CO in WHO group 3 patients. iFick-CO are represented along the y axis while the corresponding TD-CO are represented along x axis. (A) Best fit line using simple linear regression (red line) and Pearson’s correlation show the correlation between iFick and TD measurements. (B) Bland-Altman plot graphically represents the mean difference (bias) between iFick-CO and TD-CO. There is a weak agreement iFick-CO and TD-CO using Lin’s Concordance Correlation Coefficient. CCC, Concordance Correlation Coefficient; CI, confidence interval; CO, cardiac output; iFick, indirect Fick; SD, standard deviations; TD, thermodilution; WHO, World Health Organization.

Among patients with WHO group 3 PH, iFick-CO was higher than TD-CO and the measurements were significantly different (5.93 ± 1.5 liter/min vs 5.46 ± 1.28 liter/min, *p* = 0.0061). Similarly, iFick-CI was higher than TD-CI and the measurements were significantly different [2.5, IQR (2.5-3.4) liter/min/m^2^ vs 2.32 (2.31-3.11) liter/min/m^2^, *p* = 0.0078). Regression analysis revealed a weak strength of agreement between the 2 measurements (Pearson’s rho = 0.2916, *p* value = 0.0039). Bland-Altman plots revealed a very weak agreement between iFick-CO and TD-CO [95% confidence interval for Lin’s CCC (0.80, 0.44)] (Figure 1). Unlike WHO group 3 PH, the mean TD-CO was higher than the mean iFick-CO in WHO group 1 PH, but the difference was not significantly different (5.34 ± 1.64 vs 4.77 ± 1.86 liter/min, *p* = 0.087). Among WHO group 3 PH, there were no significant differences between CO and CI values measured by iFick or TD among COPD and ILD patients (data not shown).

In WHO group 3 patients, TD-PVR was higher than iFick-PVR [3.1 (IQR 2.04-3.75) WU vs 2.87 (IQR 1.98–3.69) WU, *p* = 0.026]. TD-PVR was associated with the need for CBP (*p* = 0.0001), and 1- and 3-month mortality post-transplant (*p* = 0.0001 and *p* = 0.005, respectively). iFick-PVR is associated with the need for CBP (*p* = 0.0003) and 1-month mortality (*p* = 0.0165) but not the 3-month mortality post-transplant (*p* = 0.13) (Table 2). TD-PVR shows stronger statistical significance across multiple outcomes, particularly CBP (*p* = 0.0001) and 1-month mortality (*p* = 0.0002), as compared to iFick-PVR for the same outcomes (CBP: *p* = 0.0005, 1-month mortality: *p* = 0.0201). The R-squared values for TD-PVR were consistently higher for outcomes such as 1-month mortality (R^2^ = 0.1450 for TD-PVR vs 0.0599 for iFick-PVR) and CBP (R^2^ = 0.1482 for TD-PVR vs 0.1299 for iFick-PVR).

**Table 2:** Relationship Between Post Lung Transplant Outcomes and TD-PVR and iFick-PVR

Measurement/method	Post-transplant outcomes	Estimate	R-square	*p* value
TD-PVR				
	ECMO preop	−0.04	0.0000	0.9902
	Intra OP	−0.29	0.0024	0.6361
	Post OP	−0.16	0.0004	0.8420
	CBP	2.44	0.1450	0.0001
	Need for iNO	0.62	0.0109	0.3124
	PGD	0.04	0.0002	0.8905
	MV days	0.003	0.0002	0.9007
	Tracheostomy	0.45	0.0054	0.4749
	ICU (days)	0.002	0.0001	0.9362
	LOS (days)	0.01	0.0018	0.6822
	1-Month mortality	−7.88	0.1436	0.0001
	3-Month mortality	−4.90	0.0792	0.0055
	12-Month mortality	1.20	0.0125	0.2783
				
iFick-PVR				
	ECMO preop	0.09	0.0000	0.9762
	Intra OP	−0.43	0.0058	0.4632
	Post OP	−0.01	0.0000	0.9906
	CBP	2.22	0.1289	0.0003
	Need for iNO	0.59	0.0104	0.3239
	PGD	0.07	0.0006	0.8060
	MV days	0.01	0.0017	0.6901
	Tracheostomy	0.39	0.0043	0.5248
	ICU (days)	0.001	0.0007	0.7932
	LOS (days)	0.01	0.0027	0.6163
	1-Month mortality	−4.90	0.0596	0.0165
	3-Month mortality	−2.56	0.0242	0.1300
	12-Month mortality	0.79	0.0059	0.4583

CBP, cardiopulmonary bypass; ECMO, extracorporeal membrane oxygenation; ICU, intensive care unit; iFick, indirect Fick; iNO, inhaled nitric oxide; LOS, length of stay; MV, mechanical ventilator; OP, operative; PGD, primary graft dysfunction; PVR, pulmonary vascular resistance; TD, thermodilution.

## Discussion

To our knowledge, this is the first study to compare the iFick-CO and TD-CO in patients with WHO group 3 PH. We found a weak correlation between iFick-CO and TD-CO but there was a significant difference between the 2 COs, and iFick-CO was higher than TD-CO in WHO group 3 PH. The iFick-CO and TD-CO were significantly different in WHO group 1 PH validating our findings. Similarly, the PVRs calculated using iFick-CO and TD-CO were also significantly different among the WHO group 3 PH cohort. The differences between iFick-PVR and TD-PVR were more pronounced in WHO group 3 patients when compared to WHO group 1 patients. We also observed that TD-PVR had a significant association with measures of poor outcomes after lung transplant: the need for CBP and 1- and 3-month mortality rates.

Previously, multiple studies have described discrepancies between TD-CO and iFick-CO. In a retrospective study of 300 elderly undergoing RHC, the predictive value of iFick-CO in a single patient was found to be low and not as accurate as TD-CO in the real-world setting.^20^ In 198 PAH patients, Fares et al found that 43% of the patients had a more than 20% difference in the 2 COs across patients with or without PH, suggesting an inherent discrepancy between TD and Fick independent of the characteristics of the population or hemodynamics.^21^ Similarly, Opotowsky et al noted that in a large cohort of 15,000 US veterans who underwent RHC, estimated Fick and TD-CO measurements agreed poorly, differing by >20% in over one-third of patients.^22^ There is little information on the comparison of TD and Fick methods in patients with chronic lung diseases. In patients undergoing RHC for dyspnea, COs measured by TD, and indirect and direct Fick methods differed significantly.^23^

The poor agreement of TD-CO and iFick-CO is likely due to inaccuracy introduced by the calculation of VO_2_ using the Lafarge Miettinen equation.^24^ It is the most common equation used to calculate estimated VO_2_ and is known to over-estimate VO_2_, which explains why, unlike previously published studies, iFick-CO was higher than TD-CO in our WHO group 3 patients.^25^^,^^26^ The equation was developed by studying healthy individuals between 3 and 40 years who were under anesthesia and mechanically ventilated. Therefore, the formula may not be applicable to older individuals or in disease states. Resting VO_2_ is lower in patients with heart failure and inversely proportional to the New York Heart Association class.^27^ The Lafarge Miettinen equation has not been systematically studied in patients with lung diseases, so its accuracy in calculating assumed VO_2_ in patients with chronic lung diseases is not known. The VO_2_ is influenced by many variables, including age, height, heart rate, maximum minute ventilation, obesity, and general conditioning.^28^, ^29^ Maximum minute ventilation is generally lower in patients with chronic lung disease, and these patients tend to have significant muscle wasting,^30^ which may also explain why the disagreement between iFick-CO and TD-CO was more pronounced in patients with WHO group 3 PH when compared to patients with WHO group 1 PH. However, this analysis is probably limited due to the small sample size of WHO group 1 patients.

While several studies have described differences between COs measured using different techniques, it is unclear as to which of the measured COs best predicts disease severity and outcomes. Our study included patients with advanced lung diseases who successfully underwent lung transplantation. This allowed us to study the association between COs and lung transplant outcomes. Lung transplantation in patients with PH is associated with worse outcomes due to pretransplant right ventricular dysfunction and post-transplant development of left ventricular dysfunction. In these patients, there is increased use of mechanical circulatory support perioperatively and a higher incidence of PGD and mortality in the first year. While iFick-PVR and TD-PVR had significant association with CBP and 1-month mortality rates, only TD-PVR had a significant association with 3-month mortality rates. Given the consistently stronger *p* values and R^2^ values for TD-PVR across these outcomes, the data suggest that TD-PVR may have a more robust predictive relationship with lung transplant outcomes than iFick-PVR. Similarly, in a large cohort of US veterans who underwent RHC, authors noted that the TD-CI better predicts mortality and should be favored over iFick in clinical practice.^22^ The ERS/ESC 2022 PAH guidelines also recommend that TD-CO should be the method to be utilized over iFick-CO (unless intracardiac shunt is suspected).^1^

The implications of our findings are significant. The CI is incorporated into the composite allocation score which determines the listing status for lung allocation. Hence, inaccurate calculation of CO has the potential to affect lung transplant listing status. Further, inaccurate CO may result in miscalculation of PVR, which is an important prognosticator in WHO group 3 PAH and guides treatment plans.

Our study has significant limitations. This was a retrospective chart review performed in a single center. Single-center study designs often lack diversity and may not accurately represent the population outside the study area. Additionally, the cohort size was modest, and the study was not powered to study all the major outcomes after lung transplant.

## Conclusion

COs measured by iFick and TD methods can be significantly different in patients with WHO group 3 PH. Our study indicates that in cases of discrepancy, PVR calculated using TD-CO correlates better with clinical outcomes in patients with WHO group 3 PH who undergo lung transplantation. Larger studies across multiple centers are needed in the future to validate these findings.

## Ethical approval

The study was conducted with IRB approval.

## CRediT authorship contribution statement

Komal Alam, MPH – Wrote the manuscript; Jose Rivera-Robles, MD – Wrote the manuscript; Audrene Edwards, MS – Performed statistical analysis; Ahmad Abdelreheim, MD – Performed retrospective chart review to obtain data for analysis; Ahmed Abdelmonem, MD – Performed retrospective chart review to obtain data for analysis; Susan Mathai, MD – Study design, Manuscript editing; Michael D Duncan, MD – Study design, Manuscript editing; Chetan Naik, MD, MS – Principal investigator conceived and presented the idea, supervised drafting of the manuscript, and finalized the manuscript.

## Disclosure statement

None of the authors have conflicts of interest related to the study topic.

Acknowledgments: None.

This research was unfunded.

Guarantor: Chetan Naik, MD, MS, accepts full responsibility for the work and/or the conduct of the study, has access to the data, and controls the decision to publish.
